# Case Report: Autoimmune glial fibrillary acidic protein astrocytopathy complicated with Sjogren’s syndrome and recurrent respiratory failure

**DOI:** 10.3389/fimmu.2025.1669415

**Published:** 2025-10-01

**Authors:** Po-You Chen, Tzu-Han Lee, Wan-Lun Tsai, Hua-Ren Chang, Kuo-Sen Tseng, Cheng-Che Wu

**Affiliations:** ^1^ Department of Physical Medicine and Rehabilitation, Taoyuan General Hospital, Ministry of Health and Welfare, Taoyuan, Taiwan; ^2^ Department of Neurology, Taoyuan General Hospital, Ministry of Health and Welfare, Taoyuan, Taiwan; ^3^ Department of Rheumatology, Taoyuan General Hospital, Ministry of Health and Welfare, Taoyuan, Taiwan

**Keywords:** autoimmune glial fibrillary acidic protein astrocytopathy, acute disseminated encephalomyelitis, Sjogren’s syndrome, multidisciplinary rehabilitation training, case report

## Abstract

Autoimmune glial fibrillary acidic protein (GFAP) astrocytopathy (GFAP-A) is a recently identified autoimmune encephalitis. We reported a case of a male in his 50s with autoimmune GFAP-A complicated with Sjogren’s syndrome and recurrent respiratory failure. The patient presented with acute and disabling encephalitis and myelitis, with symptoms including respiratory failure, swallowing dysfunction and limbs weakness. Autoimmune tests showed elevated GFAP and positive antinuclear antibody, anti-Ro and anti-Smith antibodies. MRI revealed longitudinal hypersignal from the anterior medulla to the C1 spinal cord. The clinical symptoms were favorably improved by steroid treatment and rehabilitation training. This case highlighted the spectrum of clinical manifestations associated with GFAP-A. Our findings also supported the effectiveness of rehabilitation training in treating this disease. Further investigation regarding diagnostic criteria, imaging characteristics, and the role of rehabilitation training in treating GFAP-A is necessary. This case was presented herein to shed more light on various aspects of this disease.

## Highlights

- Glial fibrillary acidic protein (GFAP) astrocytopathy is a rare but increasingly recognized autoimmune disease with variable neurological presentations, including encephalomyelitis and seizures.
- Brainstem involvement may result in respiratory failure requiring intensive care, particularly if the medulla is affected.
- Coexisting systemic autoimmune diseases such as Sjogren’s syndrome may be present and should be considered in atypical presentations.
- GFAP astrocytopathy often responds well to corticosteroid treatment.
- Multidisciplinary rehabilitation, including swallowing therapy, can significantly improve functional outcomes.

## Introduction

Autoimmune glial fibrillary acidic protein astrocytopathy (GFAP-A) is a novel central nervous system autoimmune inflammatory disease defined in 2016 (1). GFAP-A presents with a combination of symptoms involving the brain parenchyma, meninges, spinal cord, optic nerves, and peripheral nerves (2–4). The disease is commonly diagnosed in individuals aged over 40 and most patients have an acute or subacute onset (2, 5). Clinical manifestations include preceding flu-like symptoms, encephalitis (delirium, seizure, psychiatric symptoms, tremor), meningitis (headache, neck stiffness, vomiting), myelitis (paresthesia, weakness, bladder dysfunction), peripheral neuropathy (numbness) and blurred vision (6–8). A distinctive MRI feature is the perivascular radial gadolinium enhancement in the brain’s white matter, along with longitudinal enhancement in the spinal cord (9, 10). A co-occurring neoplasm, most often an ovarian teratoma, is present in approximately 25% of cases (11). While most patients only experience the condition once, relapses occurs in about 20% of GFAP-A which require long-term medication to manage without steroids (9). The detection of IgG antibodies against GFAP in the CSF, along with classic clinical presentations of the disease, serve as indicators of GFAP-A.

Rehabilitation is practiced to improve function in patients with central nervous system disorders such as multiple sclerosis. Individualized multidisciplinary rehabilitation program should focus on improving functional recovery and quality of life, basing on each patient’s symptoms and goals. Rehabilitation has been used to treat GFAP-A with some positive results; however, no randomized clinical trial was conducted.

Here, we reported a unique case of GFAP-A complicated with Sjogren’s syndrome and unusual complication of recurrent respiratory failure which have not been reported before, shedding light on its unique characteristics and contributing to the knowledge on this disease. The benefits of rehabilitation for this disease haven’t been extensively explored in the past. Hence, our report also demonstrated the benefits of rehabilitation for his disease. This report is aimed to elucidate the specifics of GFAP-A in the context of a unique case, thereby paving the way for improved diagnostic, treatment, and rehabilitation strategies.

## Case description

A male patient in his 50s, with a medical history of hypertension, diabetes mellitus, and dyslipidemia, was under medications for these conditions. He has no family history of rheumatological or oncological conditions, except diabetes mellitus.

The patient experienced rapidly progressing weakness of right extremities, leading to a significant decrease in his mobility and dependence on assistance for daily activities. He also reported numbness in distal ends of both upper extremities, episodes of simple partial seizures, and urinary retention. There were no symptoms of fever, upper respiratory issues, headache, neck pain, vomiting, blurred vision, or diplopia.

**Figure 1:**
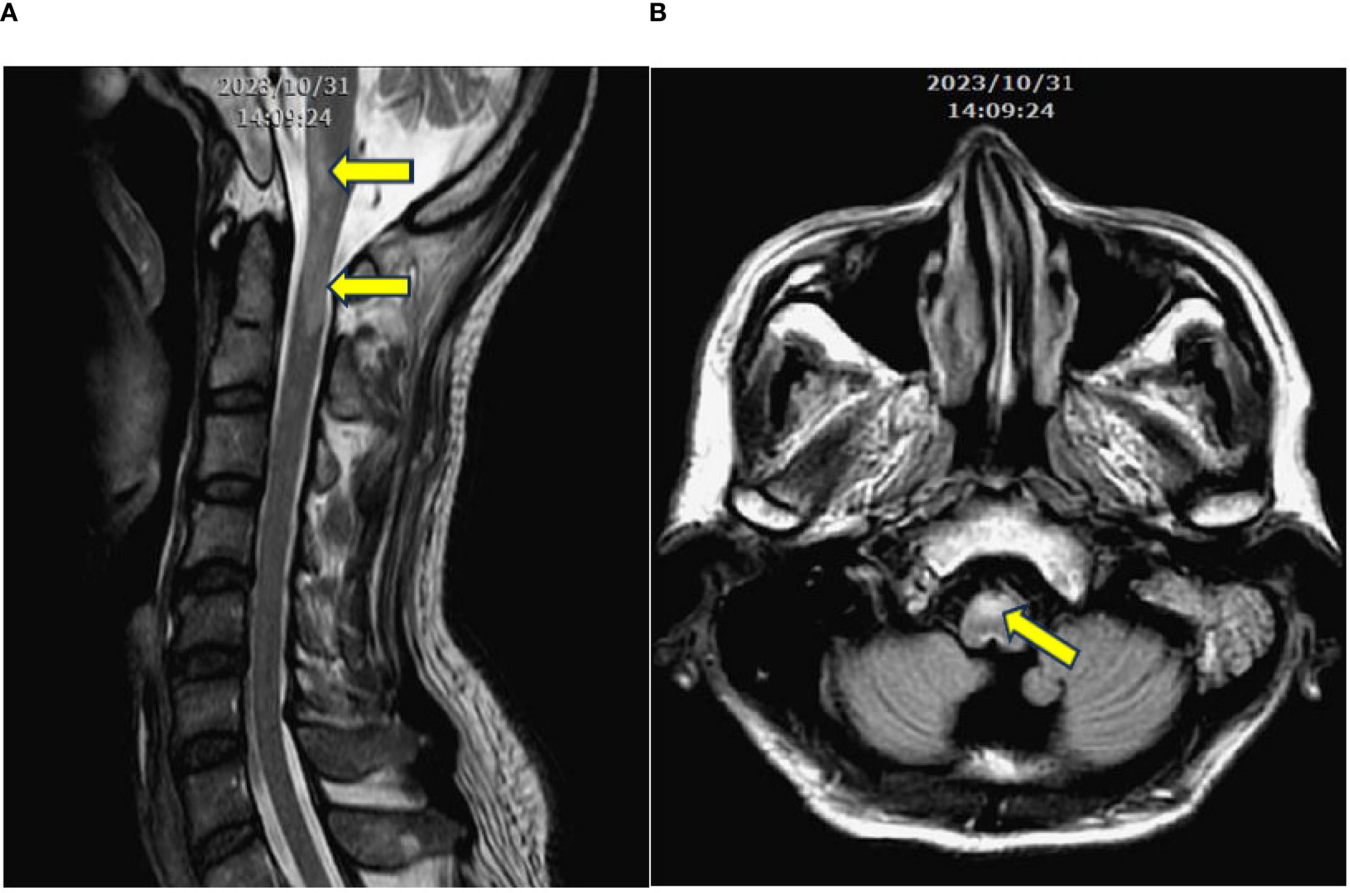
**(A)** Contrast-enhanced T2-weighted MRI in the longitudinal view, revealing a longitudinal signal increase extending from the level of medulla to C1. **(B)** Signal increase at the anterior portion of the medulla (arrows).

The patient was admitted to a hospital in Southeast Asia, and a contrast-enhanced brain MRI revealed nonspecific edematous change. A T2-weighted image revealed longitudinal hypersignal from anterior medulla to C1 spinal cord. (**Figure 1**) A CSF examination for infection returned negative results.

The patient’s condition was complicated by two episodes of acute respiratory failure with pneumonia, receiving endotracheal intubation. He also developed acute kidney injury and acute decompensated heart failure, with episodes of atrial fibrillation. The patient was treated with methylprednisolone (1 gm QD) then hydrocortisone (300 mg IV QD), empirical antibiotic meropenem for infection control, and levetiracetam injection for seizure control.

Giving the tentative diagnosis of acute disseminated encephalomyelitis (ADEM), the patient was transferred back to ICU of our hospital with a state of conscious drowsiness, a shallow breathing pattern, decreased 4 limbs muscles power (upper extremities: 3; lower extremities: 2), and bed-ridden status. During admission, the patient developed another episode of acute respiratory failure with pneumonia, necessitating intubation. Episodes of simple partial seizures in all four extremities were also observed. A cerebrospinal fluid (CSF) study showed normal protein, LDH, RBC, WBC, and cytology values, with mildly elevated glucose. A new episode of encephalomyelitis was unlikely.

Due to unexplained encephalomyelitis, an autoimmune serum examination was conducted to rule out neuromyelitis optica spectrum disorders (NMOSD) or myelin oligodendrocyte glycoprotein antibody-associated disease (MOGAD). Final serum results revealed elevated glial fibrillary acidic protein, without elevation of anti-AQP4 or anti-MOG.

**Figure 2:**
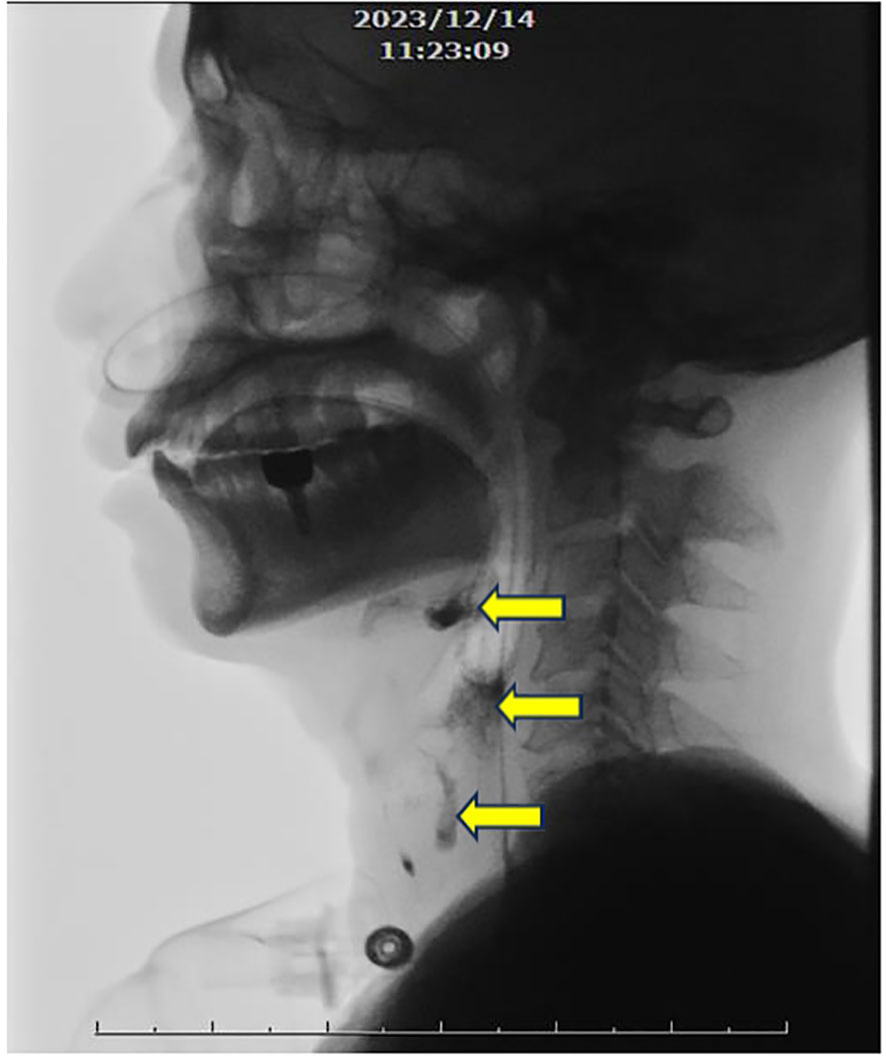
Videofluroscopic swallowing study. Weak pharyngeal contraction with evident contrast retention in the valleculae and piriform sinuses after swallowing, accompanied with laryngeal penetration (arrows).

After stabilizing medical conditions, he was transferred to rehabilitation ward with decreased muscle power of 4 limbs with 3 scores in manual muscle test (MMT), fair trunk stability and sitting balance, disability to stand up from sitting, near totally dependent ADL, and dysphagia. Rehabilitation program was arranged, including muscle strengthening and endurance training for 4 limbs weakness, balance and trunk stability training, ambulation training, functional training, ADL training, and swallowing training for dysphagia. Video-fluoroscopy of swallowing, VFSS, was performed, revealing weak pharyngeal contraction with contrast retention in the valleculae and piriform sinuses after swallowing, accompanied with laryngeal penetration (**Figure 2**). Swallowing training with pharyngeal muscles strengthening and swallowing technique was performed for dysphagia and laryngeal penetration.

After treatment for one month, the patient developed numerous itchy skin rashes. A positive antinuclear antibody was noted, along with elevated ESR. Anti-Ro and anti-Smith antibodies were also positive, while anti-DNA Ab, lupus anticoagulant, anti-Scl-70, C3, and C4 remained within normal ranges. Suspecting Sjogren’s syndrome, a Schirmer’s test was arranged and the result was positive. Steroids and azathioprine were prescribed for Sjogren’s syndrome management. Steroids were later tapered and azathioprine was continued for management of Sjogren’s syndrome and GFAP-A.

After intensive rehabilitation training, the patient’s condition improved gradually. He was able to walk under supervision without device or assistance. His muscle power of 4 limbs were rated as 4 on the MRC scale. His ADL became mostly independent, the Foley catheter and nasogastric tube were removed following successful training with safe swallowing and no choking condition. The patient was satisfied with the results of multidisciplinary rehabilitation training.

## Timeline

### Diagnostic assessment

#### Investigations

Initial investigations included contrast-enhanced MRI of brain, which showed nonspecific edematous changes. A T2-weighted cervical spine MRI revealed a longitudinal hyperintense signal from the anterior medulla to the C1 spinal cord.

Cerebrospinal fluid analysis performed twice during the disease course showed normal protein, lactate dehydrogenase (LDH), red and white blood cell counts, and cytology, with mildly elevated glucose levels. No evidence of active infection or malignancy was found.

Serological autoimmune work-up revealed:

▪ Elevated serum GFAP antibodies (Titer: 248.299; normal range: < 130)
▪ Positive antinuclear antibody
▪ Positive anti-Ro and anti-Smith antibodies
▪ Normal anti-DNA antibody, lupus anticoagulant, anti-Scl-70, complement levels (C3, C4)
▪ Negative serum anti-AQP4 and anti-MOG antibodies
▪ A Schirmer’s test was positive, confirming impaired tear production.

#### Differential diagnosis

Differential diagnoses for our case, in comparison with similar cases, include infectious meningoencephalitis, most prevalently HSV infection. Infectious meningoencephalitis typically presents with fever, headache, and meningismus. CSF analysis often reveals lymphocytic predominance and RBCs in the absence of trauma. Diagnostic CSF PCR for HSV-1 is recommended, while testing for other viral pathogens would depend on travel or exposure history. Multiple sclerosis (MS) should also be contemplated as it is usually marked by recurrent demyelinating myelopathy or optic neuritis. However, the diagnosis of MS becomes less probable with the presence of encephalopathy and GFAP antibodies (12, 13). NMOSD could potentially manifest with symptoms such as optic neuritis, transverse myelitis, postrema syndrome, and the presence of anti-AQP4 antibodies, which are directly involved in the pathogenesis of NMOSD. Some highly specific symptoms for NMOSD include excessive daytime somnolence, reversible posterior leukoencephalopathy syndrome, and neuroendocrine disorders. MOG antibody-associated disorder is characterized by ADEM and other disorders, including optic neuritis, transverse myelitis, brainstem encephalitis, and positive MOG antibody (14, 15). Vasculitis could mimic ADEM, involving multifocal deficits secondary to deep brain infarction. Abnormal vessels would be revealed by magnetic resonance angiography. Vasculitis secondary to systemic autoimmune disease could be diagnosed by appropriate rheumatologic serologies.

#### Treatment

The patient received high-dose intravenous methylprednisolone (1 g daily), followed by hydrocortisone (300 mg IV daily) during acute phase. Empirical antibiotic therapy with meropenem was initiated due to suspected aspiration pneumonia. Seizures were managed with intravenous levetiracetam.

Following stabilization, the patient received a comprehensive inpatient rehabilitation program, including muscle strengthening, balance and ambulation training, functional task training, and swallowing training based on VFSS findings.

Upon diagnosis of Sjogren’s syndrome, azathioprine was added to maintain immunosuppression, and steroids were gradually tapered.

#### Outcome and follow-up

After several weeks of intensive rehabilitation and immunosuppressive therapy, the patient demonstrated significant improvement. He achieved independent ambulation without assistive devices and regained strength in all four limbs (MMT:4). His swallowing function improved, permitting safe oral intake, and both the nasogastric tube and Foley catheter were removed.

At follow-up, the patient was functionally independent in daily activities and satisfied with his recovery trajectory. Azathioprine was continued for long-term immunomodulation.

## Discussion

We presented the case of a man in his 50s diagnosed with autoimmune GFAP-A, complicated with Sjogren’s syndrome and recurrent respiratory failure. The patient also exhibited a range of associated comorbidities, including acute onset of limb weakness and numbness, seizures, a senseless and distended urinary bladder, dysphagia, acute kidney injury, and acute decompensated heart failure with atrial fibrillation. Noteworthy recovery was observed following steroid treatment and rehabilitation training.

Our case shares several clinical features with previous reports, including meningoencephalitis, myelitis, seizures, and neurological bladder (16). A positive response to corticosteroid treatment was observed in our case, suggesting the responsiveness seen in most GFAP-A cases. Additionally, our findings of the presence of multiple autoimmune antibodies are in agreement with existing reports. The classical presentation of longitudinal T2W enhancement of MRI in spinal cord, typically associated with GFAP-A (17, 18), were evident in our case.

In our case, a history of preceding flu-like symptoms was absent, which are quite common in GFAP-A (11). Additionally, we observed no presence of psychiatric symptoms, dysautonomia, or optic disc papillitis (19, 20). The CSF tests in this case showed normal levels of protein, lactate dehydrogenase (LDH), red and white blood cell counts, and cytology, as well as negative results for infectious pathology and cultures, with only a mildly elevated glucose level (121 mg/dL). Such a finding has not been commonly reported in previous studies. In a case-series study, neoplasms were detected in 22 out of 102 patients (11), however, no such findings were observed in our case. The above presentations suggest the heterogeneity of GFAP-A.

The significance and uniqueness of this case lie in the fact that GFAP-A, being a relatively newly discovered disease, still has many unexplored features. In our case, the patient experienced recurrent respiratory failure, necessitating intubation. This finding is postulated to be attributive to an attack on the medulla, where the respiratory center is located. A recent cohort study reported that respiratory failure requiring intubation was also noted in 23% of cases, with many also showing brainstem involvement (21). From our experiences in ICU care, this manifestation of medulla attack leading to intubation has also been observed in other CNS diseases. In addition to recurrent respiratory failure, we reported another unique aspect of present case as the relation to Sjogren’s syndrome. Coexisting autoimmune antibodies in serum are apparent in approximately 20% of cases (11, 21), or even up to 75% in a Chinese study (6). The most common overlapping autoimmune syndromes in patients with GFAP-A were anti-AQP4 or anti-MOG (22). However, a clear clinical presentation of Sjogren’s syndrome was noted in our study, which had not been reported before. Therefore, further exploratory studies on GFAP associated with non-neural autoimmune diseases are warranted.

Regarding the prognosis and disease prevention, approximately twenty percent of GFAP-A cases experience a relapse (9), necessitating a transition to a steroid-sparing drug. Outcomes have been reported to vary, and early and sustained intervention often leads to well recovery (9). The prognosis appears to be favorable, with a median modified Rankin score of 1 being reported (11). In milder cases, complete self-recovery may occur even in the absence of treatment (6, 23). As the pathogenesis of GFAP-A remains unclear, no preventative methods have been identified to date.

A limitation of our study was the absence of certain infectious or heavy-metal surveys at the onset of the clinical course. For example, not all potential pathogens were investigated, even though the clinical symptoms and contact history did not suggest their presence. In addition, a longer follow-up period would have been beneficial to fully comprehend this case. Despite these limitations, our study revealed a unique presentation of GFAP-A associated with recurrent respiratory failure and Sjogren syndrome, highlighting the heterogeneity of this disease.

Autoimmune GFAP astrocytopathy is a newly defined central nervous system autoimmune inflammatory disease with various phenomenon and complications. Our report delves into a specific case of GFAP-A, shedding light on its unique characteristics and contributing to the knowledge on this disease, including diagnosis, treatment, and rehabilitation strategies. Further research may be needed for more understanding on this new disease, including diagnostic criteria and effectiveness of rehabilitation.

## Patient’s perspective

According to the statement of the patient, he felt extreme fear for his condition in the beginning of disease course because he was paralytic with intubation. He felt lucky and thankful to recover well after intensive treatment and rehabilitation. He could walk independently with total independent ADL and travel everywhere now.

## Data Availability

The original contributions presented in the study are included in the article/supplementary material. Further inquiries can be directed to the corresponding author.

## References

[1] Fang B McKeon A Hinson SR Kryzer TJ Pittock SJ Aksamit AJ. Autoimmune glial fibrillary acidic protein astrocytopathy: A novel meningoencephalomyelitis. JAMA Neurol. (2016) 73:1297–307. doi: 10.1001/jamaneurol.2016.2549, PMID: 27618707

[2] Shan F Long Y Qiu W. Autoimmune glial fibrillary acidic protein astrocytopathy: A review of the literature. Front Immunol. (2018) 9:2802. doi: 10.3389/fimmu.2018.02802, PMID: 30568655 PMC6290896

[3] Iorio R Damato V Evoli A Gessi M Gaudino S Lazzaro V. Clinical and immunological characteristics of the spectrum of GFAP autoimmunity: a case series of 22 patients. J Neurol Neurosurg Psychiatry. (2018) 89:138–46. doi: 10.1136/jnnp-2017-316583, PMID: 28951498

[4] Tokimura R Matsuda N Kobayashi S Kimura A Kanai K. Abnormal evoked potentials in autoimmune glial fibrillary acidic protein astrocytopathy. eNeurologicalSci. (2020) 18:100229. doi: 10.1016/j.ensci.2020.100229, PMID: 32090177 PMC7026450

[5] Lan W Li J Ai P Luo W. Autoimmune glial fibrillary acidic protein astrocytopathy: clinical analysis and review of 15 cases. Acta Neurol Belg. (2023) 123:1465–79. doi: 10.1007/s13760-023-02268-0, PMID: 37079256 PMC10117260

[6] Long Y Liang J Xu H Huang Q Yang J Gao C. Autoimmune glial fibrillary acidic protein astrocytopathy in Chinese patients: a retrospective study. Eur J Neurol. (2018) 25:477–83. doi: 10.1111/ene.13531, PMID: 29193473

[7] Wang H Chin JH Fang BY Chen X Zhao AL Ren HT. Autoimmune glial fibrillary acidic protein astrocytopathy manifesting as subacute meningoencephalitis with descending myelitis: a case report. BMC Neurol. (2020) 20:443. doi: 10.1186/s12883-020-02021-7, PMID: 33297961 PMC7727233

[8] Yaguchi T Kimura A Takekoshi A Matsuo M Tomita H Shimohata T. Autoimmune glial fibrillary acidic protein astrocytopathy associated with breast cancer: a case report. BMC Neurol. (2023) 23:145. doi: 10.1186/s12883-023-03194-7, PMID: 37016352 PMC10071775

[9] Kunchok A Zekeridou A McKeon A. Autoimmune glial fibrillary acidic protein astrocytopathy. Curr Opin Neurol. (2019) 32:452–8. doi: 10.1097/wco.0000000000000676, PMID: 30724768 PMC6522205

[10] Tewkesbury G Song JW Perrone CM. Magnetic resonance imaging of autoimmune GFAP astrocytopathy. Ann Neurol. (2021) 90:691–2. doi: 10.1002/ana.26195, PMID: 34390019

[11] Flanagan EP Hinson SR Lennon VA Fang B Aksamit AJ Morris PP. Glial fibrillary acidic protein immunoglobulin G as biomarker of autoimmune astrocytopathy: Analysis of 102 patients. Ann Neurol. (2017) 81:298–309. doi: 10.1002/ana.24881, PMID: 28120349

[12] de Seze J Debouverie M Zephir H Lebrun C Blanc F Bourg V. Acute fulminant demyelinating disease: a descriptive study of 60 patients. Arch Neurol. (2007) 64:1426–32. doi: 10.1001/archneur.64.10.1426, PMID: 17923626

[13] Schwarz S Mohr A Knauth M Wildemann B Storch-Hagenlocher B. Acute disseminated encephalomyelitis: a follow-up study of 40 adult patients. Neurology. (2001) 56:1313–8. doi: 10.1212/wnl.56.10.1313, PMID: 11376180

[14] López-Chiriboga AS Majed M Fryer J Dubey D McKeon A Flanagan EP. Association of MOG-igG serostatus with relapse after acute disseminated encephalomyelitis and proposed diagnostic criteria for MOG-IgG-associated disorders. JAMA Neurol. (2018) 75:1355–63. doi: 10.1001/jamaneurol.2018.1814, PMID: 30014148 PMC6248120

[15] Santoro JD Chitnis T. Diagnostic considerations in acute disseminated encephalomyelitis and the interface with MOG antibody. Neuropediatrics. (2019) 50:273–9. doi: 10.1055/s-0039-1693152, PMID: 31340401 PMC7117081

[16] Qin N Wu X Wang J Wang W Wang X Ma Y. Case report: Autoimmune glial fibrillary acidic protein astrocytopathy misdiagnosed as tuberculous meningitis. Front Neurol. (2023) 14:1123603. doi: 10.3389/fneur.2023.1123603, PMID: 36970528 PMC10034075

[17] Wang S Yuan J Liu J. Autoimmune glial fibrillary acidic protein (Gfap) astrocytopa-thy accompanied with reversible splenial lesion syndrome (RESLES): A case report and literature review. Brain Sci. (2023) 13:4. doi: 10.3390/brainsci13040659, PMID: 37190624 PMC10136966

[18] Puac-Polanco P Zakhari N Jansen GH Torres C. Case 309: autoimmune glial fibrillary acidic protein astrocytopathy. Radiol Jan. (2023) 306:293–8. doi: 10.1148/radiol.211954, PMID: 36534605

[19] Greco G Masciocchi S Diamanti L Bini P Vegezzi E Marchioni E. Visual system involvement in glial fibrillary acidic protein astrocytopathy: two case reports and a systematic literature review. Neurol Neuroimmunol Neuroinflamm. (2023) 10:4. doi: 10.1212/nxi.0000000000200146, PMID: 37582612 PMC10427126

[20] Quek AM Tang D Chin A Ng KW Lin H Seet RC. Autoimmune glial fibrillary acidic protein astrocytopathy masquerading as tuberculosis of the central nervous system: a case series. Int J Infect Dis. (2022) 124:164–7. doi: 10.1016/j.ijid.2022.09.029, PMID: 36162739

[21] Gravier-Dumonceau A Ameli R Rogemond V Ruiz A Joubert B Muñiz-Castrillo S. Glial fibrillary acidic protein autoimmunity: A French cohort study. Neurology. (2022) 98:e653–68. doi: 10.1212/wnl.0000000000013087, PMID: 34799461 PMC8829963

[22] Yang X Xu H Ding M Huang Q Chen B Yang H. Overlapping autoimmune syndromes in patients with glial fibrillary acidic protein antibodies. Front Neurol. (2018) 9:251. doi: 10.3389/fneur.2018.00251, PMID: 29755396 PMC5932346

[23] Kaga M Ueda T Yoshikawa S. A rare case of glial fibrillary acidic protein astrocytopathy that resolved spontaneously within a self-limited course. Heliyon. (2023) 9:e20912. doi: 10.1016/j.heliyon.2023.e20912, PMID: 37867900 PMC10589841

